# Supplementary material for: Prevalence and associated factors of mental and substance use problems among adults in Kenya: A community-based cross-sectional study
Source: PLOS Glob Public Health. 2025 Jun 30;5(6):e0004130. doi: 10.1371/journal.pgph.0004130 (PMC12208483; doi:10.1371/journal.pgph.0004130)
Supplement: S1 Table — (DOCX) [file pgph.0004130.s001.docx]

**S1 Table. Summary of the risk and protective indicators of mental health problems among women and men**

| **Mental Health Problems** | | | | | |
| --- | --- | --- | --- | --- | --- |
| Factors associated with depressive symptoms | | Factors associated with anxiety symptoms | | Factors associated with PTSD symptoms | |
| **Women** | **Men** | **Women** | **Men** | **Women** | **Men** |
| *Risk Indicators* | *Risk Indicators* | *Risk Indicators* | *Risk Indicators* | *Risk Indicators* | *Risk Indicators* |
| - Urban residence | - Sexual abuse | - Urban residence | - Urban residence | - Urban residence | - Verbal abuse |
| - Higher educational status | - Living in rented houses | - Higher education status |  | - Primary/secondary level of education | - Somatic complaints |
| - Experiencing ≥stressful events | - Light physical activities | - Experiencing ≥5 stressful events |  | - Experiencing ≥5 stressful events | - Experiencing ≥5 stressful events |
| - Household food insecurity |  | - Poor self-reported health |  | - Household food insecurity |  |
| - Household debt |  | - Being unemployed or casual labourer |  | - Declining self-rated health status |  |
| - Poor self-reported eyesight |  |  |  |  |  |
| - Declining self-rated health status |  |  |  |  |  |
| Protective factors | Protective factors | Protective factors | Protective factors | Protective factors | Protective factors |
| - Increasing self-reported wellbeing | - increasing self-reported wellbeing | - Increasing self-reported wellbeing | - Vigorous physical activity | - Older age (>50 years) | - Increasing household income |
| - Perceived social support | - Perceived social support | - Perceived social support | - Perceived social support |  |  |
