# Supplementary material for: Prevalence and associated factors of mental and substance use problems among adults in Kenya: A community-based cross-sectional study
Source: PLOS Glob Public Health. 2025 Jun 30;5(6):e0004130. doi: 10.1371/journal.pgph.0004130 (PMC12208483; doi:10.1371/journal.pgph.0004130)
Supplement: S2 Table — (DOCX) [file pgph.0004130.s002.docx]

**S2 Table. Summary of the risk and protective indicators of substance use problems among women and men**

| Factors associated with current alcohol use | | Factors associated with any current drug use |
| --- | --- | --- |
| **Women** | **Men** | **Whole sample (men and women)** |
| Risk indicators | Risk indicators | Risk Indicators |
| - Urban residence | - Higher monthly household income | - Unemployment/casual work |
| - Experiencing ≥5 stressful events |  | - Sexual abuse |
| - Being sexually active |  |  |
| - Living in single-parent family |  |  |
| Protective indicators | Protective indicators | Protective indicators |
| - Being married/cohabiting | - Being a Muslim | - Female sex |
|  | - Experiencing multimorbidity |  |
|  | - Larger household size |  |
